# Color-tuning of natural variants of heliorhodopsin

**DOI:** 10.1038/s41598-020-72125-0

**Published:** 2021-01-13

**Authors:** Se-Hwan Kim, Kimleng Chuon, Shin-Gyu Cho, Ahreum Choi, Seanghun Meas, Hyun-Suk Cho, Kwang-Hwan Jung

**Affiliations:** 1 Department of Life Science and Institute of Biological Interfaces, Sogang University, 35 Baekbeom-Ro, Mapo-Gu, Seoul, 04107 Korea; 2 Research Center for Endangered Species, National Institute of Ecology, 23, Gowol-gil, Yeongyang-eup, Yeongyang-gun, 36531 Gyeongsangbuk-do Korea

**Keywords:** Biochemistry, Biophysics, Microbiology

## Abstract

Microbial rhodopsins are distributed through many microorganisms. Heliorhodopsins are newly discovered but have an unclear function. They have seven transmembrane helices similar to type-I and type-II rhodopsins, but they are different in that the N-terminal region of heliorhodopsin is cytoplasmic. We chose 13 representative heliorhodopsins from various microorganisms, expressed and purified with an N-terminal His tag, and measured the absorption spectra. The 13 natural variants had an absorption maximum (λmax) in the range 530–556 nm similar to proteorhodopsin (λmax = 490–525 nm). We selected several candidate residues that influence rhodopsin color-tuning based on sequence alignment and constructed mutants via site-directed mutagenesis to confirm the spectral changes. We found two important residues located near retinal chromophore that influence λmax. We also predict the 3D structure via homology-modeling of *Thermoplasmatales* heliorhodopsin. The results indicate that the color-tuning mechanism of type-I rhodopsin can be applied to understand the color-tuning of heliorhodopsin.

## Introduction

Rhodopsins have seven membrane-embedded α-helices and use retinal as their chromophore^1^. Many organisms in nature convert light energy to chemical energy by using type-I rhodopsins. In general, rhodopsins are categorized into two types: type-I (microbial rhodopsin) and type-II (animal rhodopsin)^1^. Both types of rhodopsins share similar membrane topology but have opposite chromophore isomerization upon light activation. Basically, absorbance appears to be tuned to the light available in the host’s biological niche^2^. A good example is the color-tuning of microbial rhodopsins, which exhibit variation in wavelength despite similar molecular structures. Understanding the color-tuning mechanism and modifying λmax have become an interesting basic research topic for the application of optogenetics. The outward cation-pumping rhodopsin and inward anion-pumping rhodopsin can be used to selectively hyperpolarize and inhibit action potential. Cation channel rhodopsin can be used to depolarize the neuronal cell, and anion channel rhodopsin can be used to hyperpolarize the neuronal cell. The different wavelengths of the light-absorbing property of microbial rhodopsin are thought to be very useful for optogenetics studies^3–6^.

Spectral tuning is one of the important issues in a study of rhodopsin. As rhodopsin has retinal as a chromophore, the energy gap between the ground and first excited state (S1–S0) for retinal affects the light absorption of rhodopsin. The absorption maximum (λmax) of the chromophore is 568 nm in the light-adapted form of BR, but protonated retinylidene Schiff base has a λmax of 440 nm in methanol^7^. This phenomenon is known as opsin shift and is an example of the energy gap of rhodopsin depending on the steric and electrostatic interactions between the chromophore and protein environment^8–13^. The chromophore planarity and distortion regulate S1-S0 energy and affect the spectral shift^8,14^. The electrostatic interaction induced by the charged, polar, and polarizable amino acids of the protein environment regulate the S1–S0 energy gap^12^. The chromophore environment of the retinal binding pocket contributes a lot to the color-tuning of rhodopsins^2,15,16^. The residues have also been reported to influence the color far from the retinal binding pocket^17,18^. Thus, the difference between the electronic ground state (S0) and first excited state (S1) of rhodopsin is important to understanding rhodopsin color-tuning^2,19,20^.

Recently, a new type of rhodopsin, heliorhodopsin (HeR), was discovered by functional metagenomic analysis^21^. HeR is globally distributed in a wide range of living organisms and exhibits < 15% sequence homology with type-I and type-II rhodopsins. A distinct difference between HeR and conventional rhodopsin is membrane topology^21–24^. The N-terminus of HeR is cytoplasmic, whereas type-I and type-II rhodopsin are located on the opposite side. Although differences exist in sequence similarity and orientation of the N-terminus compared to conventional rhodopsin, the molecular properties of HeR are similar to type-I rhodopsin^21^. HeR has an all-*trans*-retinal as a chromophore and all-*trans* to 13-*cis* photoisomerization upon light absorption. HeR also has a Lys residue as a protonated Schiff base. A previous study reported that HeR generates photo-intermediates K, L, M, and O during light-absorbing reactions similar to type-I ion pumping rhodopsins^21,22^. In addition, HeR has a slow photocycle^25,26^, and it seems the function of HeR is sensory reception, not ion transport^21^. A pH change was not detected upon light illumination. Thus, HeR may have light-sensory activity in such a ways that it acts as type-II rhodopsin interacts with G-protein or as type-I rhodopsin activates membrane protein or soluble protein^27^. HeRs have similar molecular properties as type-I rhodopsin. Lys in TM7 is conserved in the HeR family, retinal is attached in a Schiff base linkage to a Lys residue, and Glu residue in TM3 is conserved in the HeR family as a counter-ion^21^, which does not act as a proton acceptor for the protonation of the Schiff base. From the mutant study of His residues (H23F and H80F of 48C12 HeR), histidine residues around the N-terminus may be a proton accepting group^27^. Arg104 residue in 48C12 HeR plays a crucial role in stabilizing the protein. Met115 in 48C12 HeR is thought to be the color-tuning residue because it is consistent with the L105Q position in GPR and BPR^28^. The Ala mutation of Trp105 and Trp107 did not form rhodopsin pigment. The mutation of Ala240 to Ser and Thr resulted in blue- and red-shifts, respectively^27^.

The main objective of this study was to identify spectral tuning (concept of opsin shift) in natural variants of heliorhodopsins rather than artificial introduction of mutations. We expressed 13 new, randomly selected heliorhodopsins from various microorganisms and purified them using an Ni^2+^-NTA column. We then measured the various λmax values. In order to determine which residues influence the λmax of natural variation, we compared sequences among selected heliorhodopsins and introduced several mutations to the potential critical conserved positions. We found two residues that affect spectral tuning and tried to prove a spectral tuning mechanism based on previous type-I rhodopsin studies^29^.

## Results

### Phylogenetic analysis and measurement of absorption spectra

**Figure 1:**
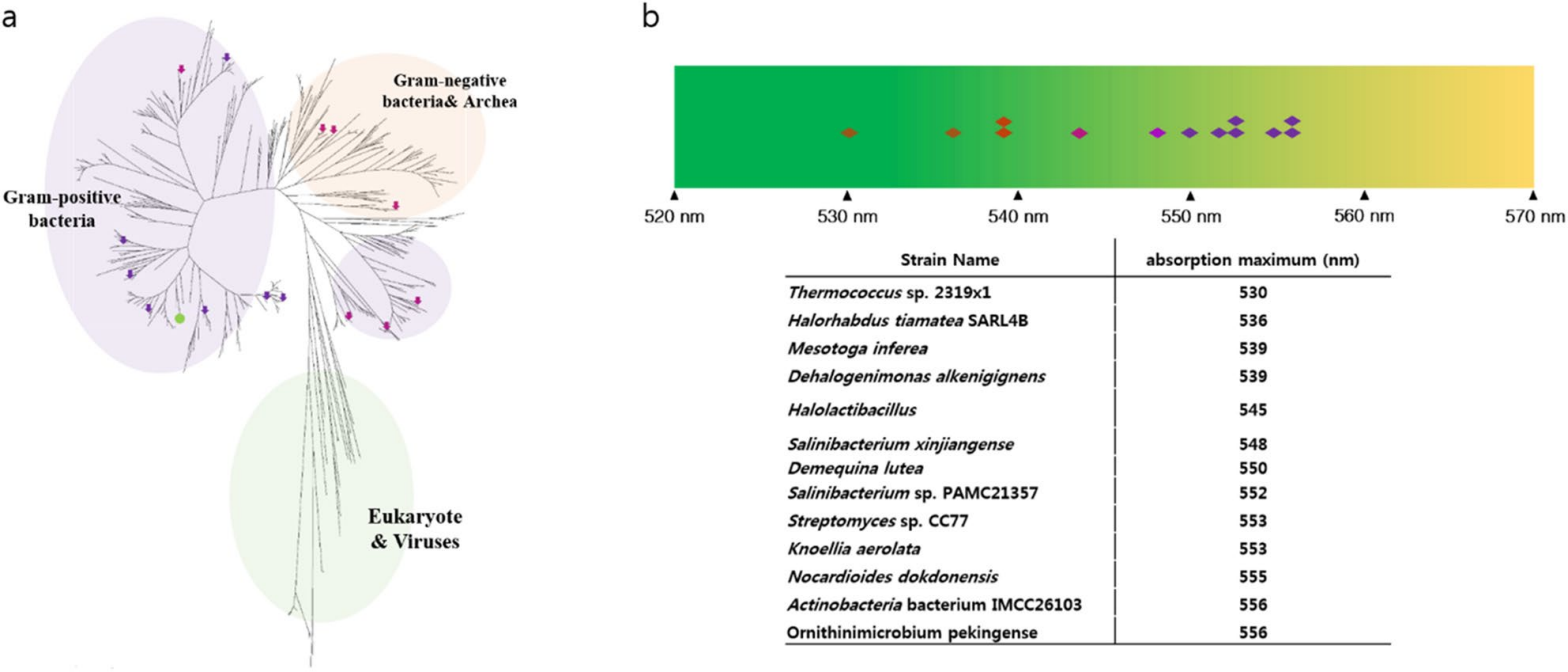
Phylogenetic tree and distribution of absorption maxima for various heliorhodopsins. (**a**) The phylogenetic tree is built with ~ 474 heliorhodopsins. Thirteen of them were selected from various microorganisms: *Thermococcus* sp. 2,319 × 1 (530 nm), *Halorhabdus tiamatea* SARL4B (536 nm), *Mesotoga* infera (539 nm), *Degalogenimonas alkenigignes* (539 nm), *Halolactibacillus* (545 nm), *Salinibacterium xinjiangense* (548 nm), *Demiquina lutea* (550 nm), *Salinibacterium* sp. PAMC21357 (552 nm), *Streptomyces* sp. CC77 (553 nm), *Knoellia aerolata* (553 nm), *Nocardioides dokdonensis* (555 nm), *Actinobacteria* bacterium IMCC26103 (556 nm), *Orinithinimicrobium pekingense* (556 nm). The arrows indicate the position of heliorhodopsins listed above, and the green circle is 48C12 HeR. Heliorhodopsins with λmax > 550 nm are marked in purple. The other heliorhodopsins are marked with pink arrows. There is a tendency for heliorhodopsins λmax > 550 nm exhibit close genetic distance. (**b**) Distribution of λmax values of various heliorhodopsins. Thirteen heliorhodopsin proteins were purified. 48C12 HeR is marked on the light spectrum. The color of the diamond represents the λmax of each HeR. In nature, the variation in λmax for heliorhodopsin is almost ~ 30 nm.

The phylogenetic tree was drawn after multiple sequence alignments (Fig. 1). The 470 heliorhodopsin protein sequences were obtained from BLAST. The HeRs from bacteria in the actinobacteria phylum formed a large clade, and HeRs from archaea and Gram-negative bacteria had close genetic distance and made up one clade. HeRs from eukaryotes and viruses made up another clade. These evolutionary analyses were described in previous reports^21,22^. Thirteen rhodopsins were randomly selected (eukaryote and virus clade is not included) and the absorption spectra for each HeR measured, with varying λmax (Fig. 1): *Thermococcus* sp. 2,319 × 1 (λmax = 530 nm), *Halorhabdus tiamatea* SARL4B (536 nm), *Mesotoga infera* (539 nm), *Degalogenimonas alkenigignes* (539 nm), *Halolactibacillus* (545 nm), *Salinibacterium xinjiangense* (548 nm), *Demiquina lutea* (550 nm), *Salinibacterium* sp. PAMC21357 (552 nm), *Streptomyces* sp. CC77 (553 nm), *Knoellia aerolata* (553 nm), *Nocardioides dokdonensis* (555 nm), *Actinobacteria* bacterium IMCC26103 (556 nm), and *Orinithinimicrobium pekingense* (556 nm). Natural variation in λmax was observed for HeR within a range of 26 nm. Unexpectedly, we found six HeRs with λmax > 550 nm that are relatively close in the phylogenetic tree. Other HeRs were spread out with a phylogenetic tree despite having similar λmax.

### Selection of residues that influence absorption spectra

**Figure 2:**
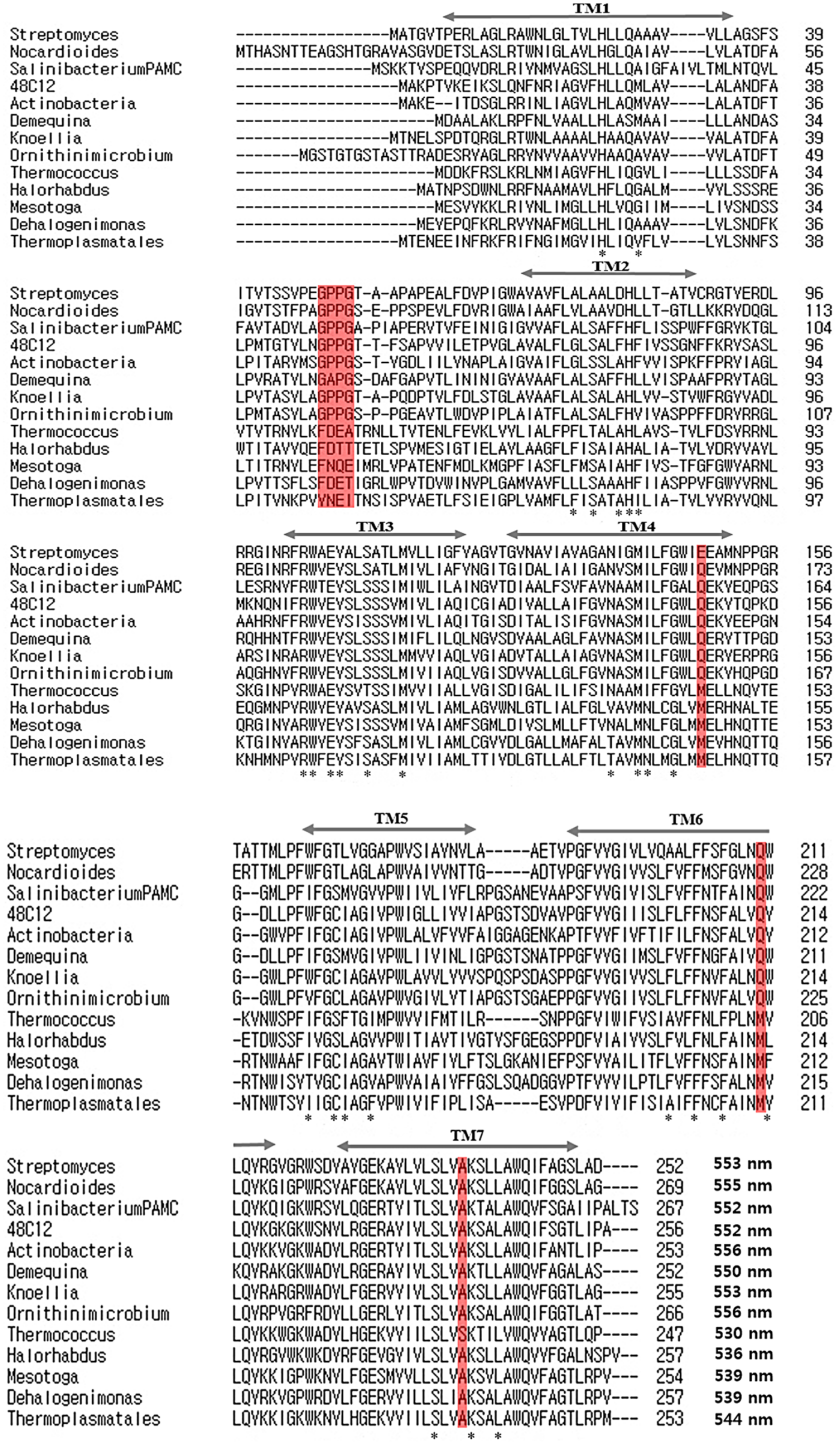
Sequence alignment of 13 heliorhodopsin sequences. Four heliorhodopsins have an absorption maximum < 540 nm, one heliorhodopsin has an absorption maximum at 544 nm, and the other heliorhodopsins have absorption maxima > 550 nm. Significantly different amino acid regions are marked in red. The asterisk (*) indicates residues that comprise the retinal binding pocket^24^.

**Figure 3:**
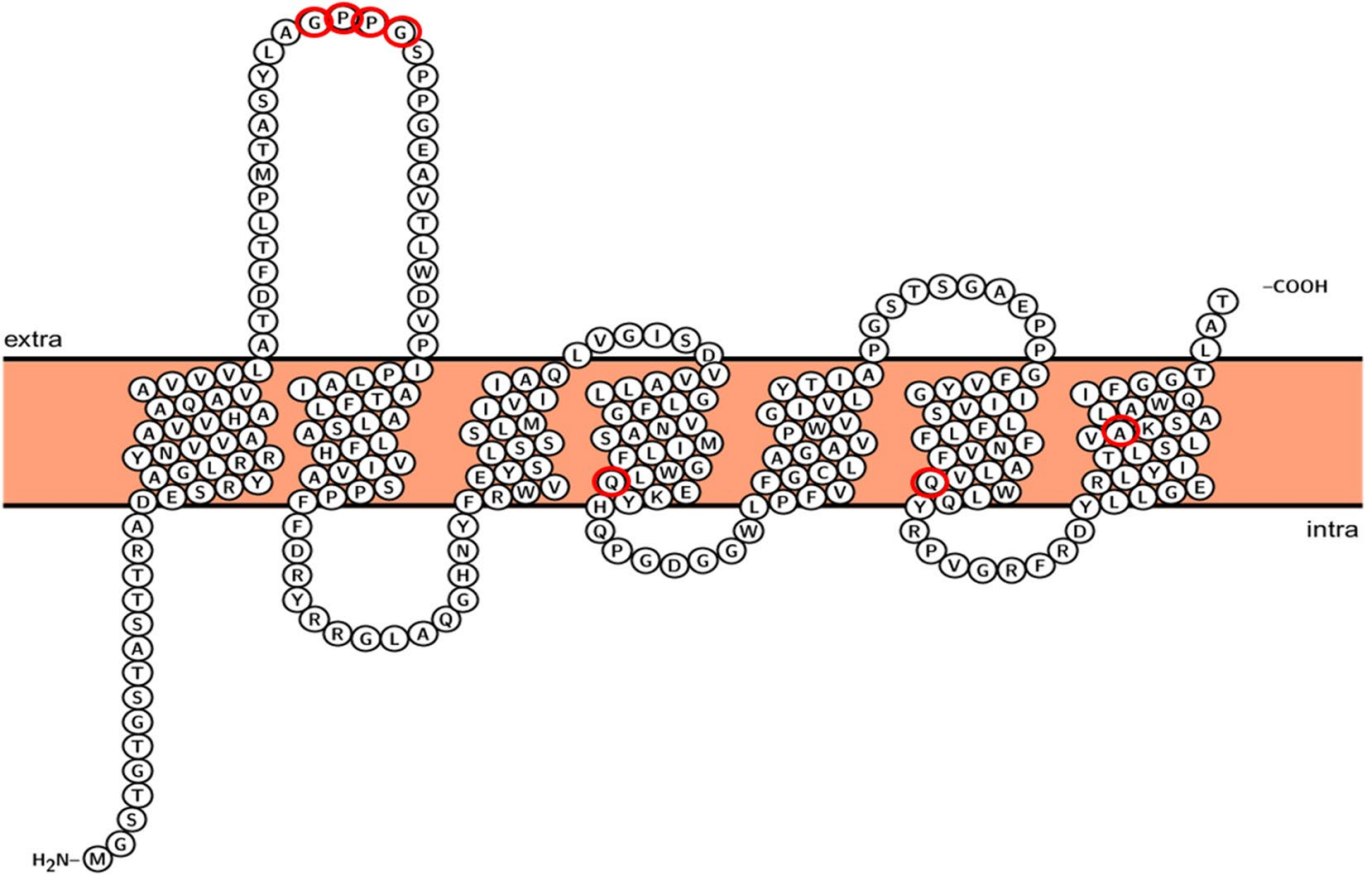
Candidates for the residues involved in the color-tuning of natural variants of heliorhodopsins. The topology prediction of *Ornithinimicrobium* heliorhodopsin (λmax = 556 nm) obtained with Protter. Red open circles are candidate amino acids for the spectral tuning site. The comparison is the sequence from Fig. 2 (G59 ~ G62, Q159M, Q224M, and A254S of *Ornithinimicrobium* heliorhodopsin) and *Thermococcus* sp. 2,319 × 1 heliorhodopsin (F44 ~ A47, M145, M205, and S232 are corresponding residues). Mutants are designed to exchange candidate residues between *Thermococcus* heliorhodopsin and *Ornithinimicrobium* heliorhodopsin.

From the results of the absorption spectra, we attempt to identify critical residues for the spectral-tuning in naturally occurring in HeRs. natural spectral-tuning of HeRs. Several studies have attempted to find the key residue for color-tuning from other type-I and type-II rhodsopins^25,28,30–33^. For instance, it is well known that single mutation L105Q (Q105L) in GPR (BPR) exhibits considerable shift in its optical absorption spectrum^28^. In order to find a residue for natural spectral-tuning, multiple sequence alignments were performed with HeRs with λmax > 550 nm and λmax < 540 nm, and analyzed the conserved residues (Fig. 2). We found distinct differences at three positions corresponding to Gln148 in TM4, Gln213 in TM6, and Ala240 in TM7 of 48C12 HeR (Fig. 3). These residues are conserved in HeRs that have λmax > 550 nm, whereas the corresponding residues for HeRs with λmax < 540 nm were replaced with Met, Met, and Ala (Ser for *Thermococcus*), respectively. To experimentally confirm the residues that influence color-tuning, we selected two HeRs from each group, *Ornithinimicrobium* heliorhodopsin (NOH, N-terminal 6 His-tagged *Ornihinimidrobium* heliorhodopsin) and *Thermococcus* sp. 2,319 × 1 heliorhodopsin (NTH, N-terminal 6 His-tagged *Thermococcus* sp. heliorhodopsin). We constructed some single mutants and combination of single mutants by swapping these residues between two HeRs (Q159M, Q224M, and A251S for NOH, M145Q, M205Q, and S232A for NTH). In addition, we constructed a mutant for four consecutive residues substituted for each other in the first extracellular loop (Fig. 3). The GPPG (Gly-Pro-Pro-Gly) of NOH and FDEA (Phe-Asp-Glu-Ala) of NTH were exchanged and we measured the absorption changes in each mutant for two heliorhodopsins.

### Measurement of absorption spectra and prediction of mutant molecular structures

The absorption spectra of WT and mutant of NOH and NTH was measured with a UV–Vis spectrophotometer. The WT NOH had a λmax of 556 nm. The Q145M of NOH did not exhibit any significant changes in absorption. The GPPG to FDEA region of NOH had a 2 nm red-shift. Therefore, these residues are less likely to contribute to the natural spectral tuning of HeR. On the other hand, Q224M and A251S exhibited a spectral blue-shift, 5 nm for each mutant. In addition, combination mutant Q224M/A251S had a 12 nm blue-shift. For the measurement of NTH absorption, a spectral shift to NOH measurements was observed. The WT NTH has a λmax of 530 nm. This is different from the NOH measurement, as all NTH mutations exhibit a minimum red-shift of 3 nm. The M205Q of NTH had the greatest red-shift (9 nm) among the NTH mutants, and the S232A of NTH had a 6 nm red-shift. Double mutant M205Q/S232A had a 12 nm red-shift, the same as a double mutant of NOH. However, the mutation in the extracellular loop did not exhibit a significant spectral shift. It had a 3 nm shift with GPPG of NTH and 1 nm shift with FDEA of NOH. We simulated the 3D structure of the chromophore in protein using SWISS-MODEL and visualized the model with PyMol. In the prediction, the distance for both sides of the retinal chromophore (Schiff base side and β-ionone ring side) was changed with the mutant (Fig. 5). For the NOH, the distance between the β-ionone ring and Q224 was longer when Gln was changed to Met. On the other hand, the distance of the Schiff base side of the chromophore was shorter when Ala was changed to Ser. In the prediction of NTH, this happens the opposite way.

## Discussion

Studying the spectral tuning of rhodopsin is important for understanding the interaction between the chromophore and apo-protein of rhodopsin, providing fundamental knowledge to clarify the molecular properties of rhodopsin. Due to the recent development of optogenetics, demand for understanding the color-tuning of rhodopsin is increasing. Several artificial molecular modifications were made to obtain the spectral shift of rhodopsins^2,18^. From research into type-I and type-II rhodopsin color-tuning, the mechanism and crucial amino acids related to color-tuning were confirmed. Strong or weak electrostatic interaction with the counter-ion causes a spectral blue- or red-shift in type-I and type-II rhodopsins, respectively^25^. The S0 and S1 of retinal are also important factors for the color-tuning of rhodopsin in type-I rhodopsins^2,19,34^. If the S1–S0 energy gap is increased, the direction of the spectral shift is blue. In contrast, there is a red spectral shift when the S1–S0 energy gap is decreased. Four factors have been reported to affect the S0–S1 energy gap^2,14,20,35–39^: conjugation of the retinal chromophore^14,35^, polarity of the retinal-binding cavity^20,36^, chromophore positioning effects^37^, and interaction between Schiff base and the Schiff base conterion^38,39^. The spectral shift of the rhodopsin is the result of a complex phenomenon that cannot be explained by only one reason. However, mutations, such as changes in the π-conjugation of the chromophore, are not a frequent event, and it may not be appropriate to describe the spectral tuning of natural variants of HeRs in this study. On the other hand, changes in the electrostatic interactions between the chromophore and protein environment could be explained as opsin shift^8–13^, and could be the reason for spectral tuning of natural variants in this study. Differences in the amino acid array shown for the natural variants of HeRs result in a difference in electrostatic interactions with chromophore.

**Figure 4:**
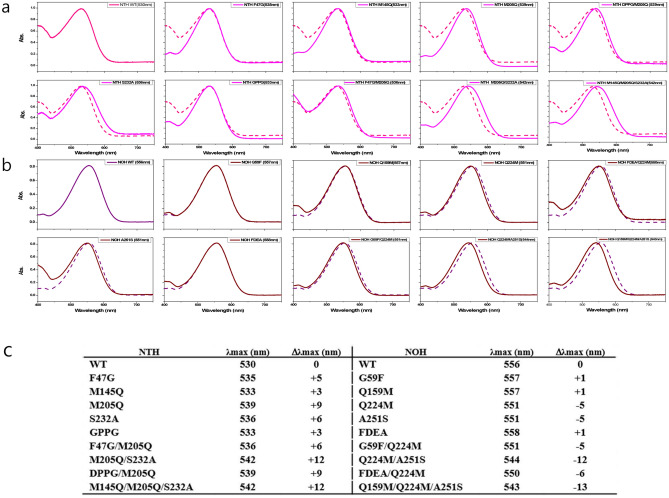
Absorption spectrum of N-terminal His-tagged *Thermococcus* heliorhodopsin (NTH) and N-terminal His-tagged *Ornithinimicrobium* heliorhodopsin (NOH). (**a**) The maximum absorption of WT and mutant NTH is between 530 and 542 nm. The double mutant (M205Q/S232A) showed a large shift to red (12 nm). The WT NTH absorption spectrum is shown as a dotted-line in the mutant absorption spectrum. (**b**) The maximum absorption of WT and mutant NOH is between 543 and 556 nm. The double mutant (Q224M/A251S) showed a large shift to blue (12 nm). The WT NOH absorption spectrum is shown as a dotted-line in the mutant absorption spectrum. (**c**) Table of λmax values for each WT and mutant heliorhodopsin.

**Figure 5:**
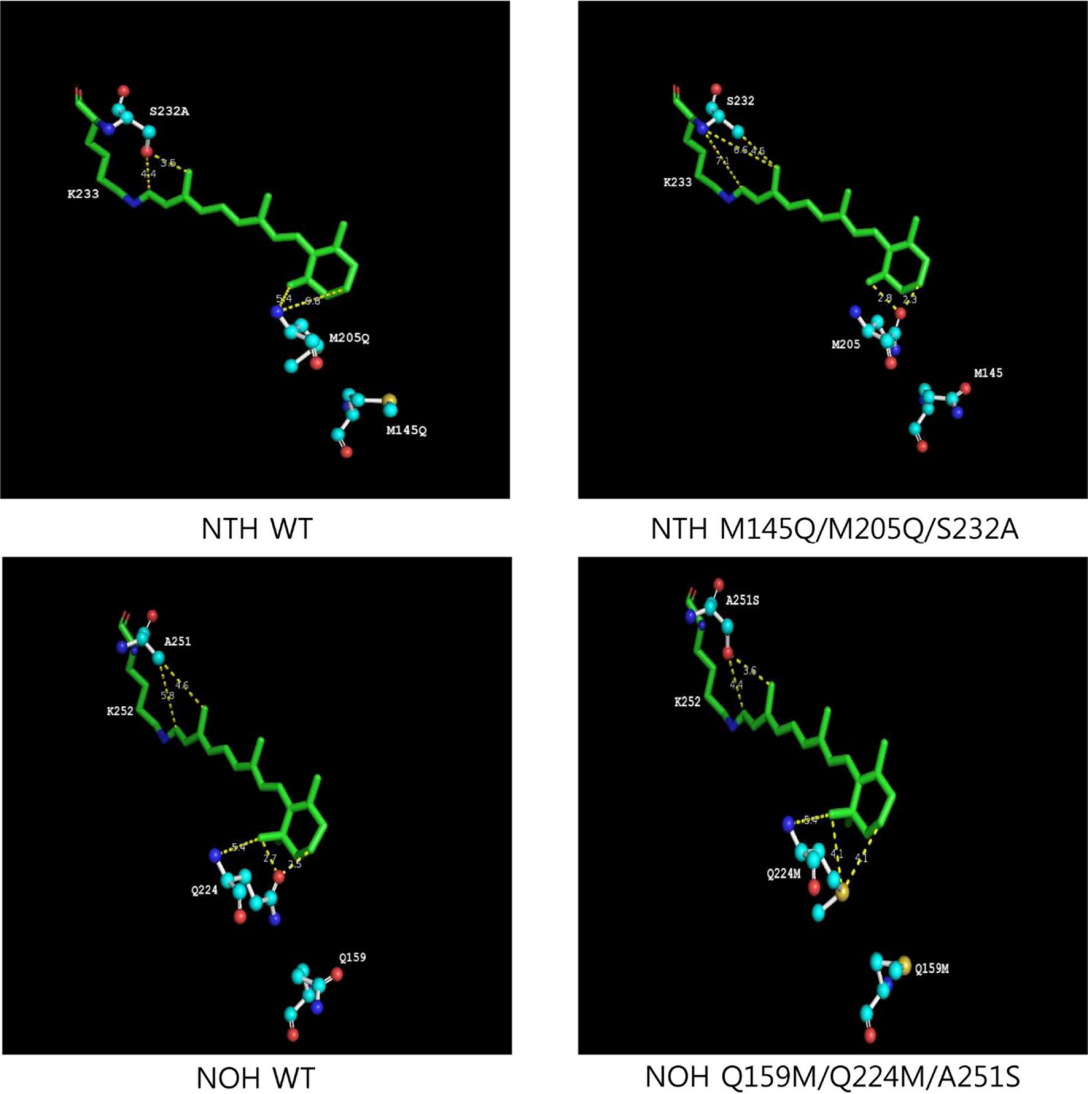
Local view of the residues involved in spectral tuning in N-terminal His-tagged *Thermococcus* heliorhodopsin (NTH) and N-terminal His-tagged *Ornithinimicrobium* heliorhodopsin (NOH) with computer modeling. The distance on both sides of the retinal chromophore (Schiff base side and β-ionone ring side) is changed along with mutant. For the NOH, the distance between β-ionone and Gln224 is greater when the Gln is replaced with Met. However, the distance of the Schiff base side of the chromophore becomes shorter when Ala is replaced with Ser. In the case of NTH, the prediction is reversed. Chromophore retinal is green, the amino group is blue, the O atom of COO^-^ is red, the C atoms of amino acids are cyan, and S-methyl is yellow.

In this study, we expressed various heliorhodopsins and tried to find the spectrally important residue of HeRs. Interestingly, the variation in λmax was 26 nm and similar to the proteorhodopsin shift (Fig. 2). Four positions in the phylogenetic tree were selected as candidates for spectral-tuning through sequence comparison. Two HeRs were selected and purified (NOH and NTH) to verify the influence of these sites. NTH had the greatest blue λmax (530 nm) and NOH had the greatest red λmax (556 nm). As we focused on finding the residues for natural spectral tuning, we replaced the corresponding residue of each HeR and measured absorption changes (Fig. 4). Consequently, Q224M and A251S of NOH had a 5 nm blue-shift with each mutant, and double mutants had a 12 nm blue-shift. On the other hand, M205Q and S232A of NTH had 9 nm and 6 nm red-shifts, respectively, and the double mutants had a 12 nm red-shift. The absorbance shift seen in double mutants is a little bit different from the sum of the changes in single mutations, as two sites do not seem to independently affect the absorption change. The predicted chromophore and protein interaction areas were located around the chromophore (Fig. 5); one is close to the Schiff base, and the other is near theβ-ionone ring. Previous studies of HeR have shown that the amino acid in front of the Lys in TM7 affects a spectral shift^27^. The A240S of 48C12 HeR also had a 5 nm blue shift in the previous study^27^. The corresponding residue for Q224 NOH is Q213 48C12 HeR, which had a blue shift. Although the replaced amino acid is different, we could confirm that the position is related to the color tuning. Involvement of the chromophore surrounding amino acids for spectral tuning was identified by the KR2 spectral-tuning study^29^. They also suggested determining the quantitative influence of amino acids surrounding the chromophore by QM/MM analysis. Our observation in this study can be interpreted with a similar mechanism of KR2 spectral-tuning^29^. First, a dipole moment is produced when A251 of NOH is replaced with Ser, increasing the S0–S1 energy gap and blue-shift of NOH absorbance. The Q224M mutant of NOH may remove a dipole moment between the β-ionone ring of the chromophore and this residue. Because the dipole moment that creates the red spectral shift disappears, the Q224M of NOH had a blue-shifted λmax. The electrostatic effects combined with double mutant Q224M/A251S of NOH resulted in a large blue-shift, but the amount of shift is not equal to the sum of shifts by single mutants. This may be the result of the chromophore positioning effect. Due to the mutations, the cavity between chromophore and surrounding amino acids can also change. In the case of NOH, this change may reduce the planarity of the chromophore and result in more blue-shift. We could adapt this mechanism for spectral changes in NTH mutants. In addition, M205T mutant of NTH had a 5 nm red-shift (data not shown). In the KR2 study, P219T exhibited a red-shift effect because the OH in the side chain reduced the S0–S1 energy gap^29^. We examined whether the OH group of HeR influenced the red shift with M205T of NTH. Through these observations, we concluded that two positions are very important for spectral tuning of the natural variations in HeRs.

Briefly, we observed variation in the λmax of HeR and identified the residues that influence the chromophores near amino acids in TM6 and TM7. As described previously, the main focus of this study was the identification of residues involved in color-tuning natural variants. A significant spectral shift of 50 nm could occur with the introduction of intentional mutation based on computational prediction, but the amount of spectral change may not be necessary for the host in nature. Therefore, we tried to find the factors that affect the spectral differences in naturally occurring HeRs by comparing the conserved residues. We confirmed that interactions between the chromophore and amino acids close to the chromophore could affect spectral tuning. Although HeR has low sequence similarity to type-I and type-II rhodopsin and different helix topology, we could confirm that the mechanism (opsin shift) of spectral tuning in other rhodopsins can be adopted to explain spectral tuning of HeR. Thus far, the function of HeR is still unknown. We expect this kind of study to be the basis of understanding the molecular mechanism of color-tuning and provide some clue to the residues needed for the research of structure and function of the HeRs.

## Methods

### Sample preparation, expression, and purification of HeRs

We randomly selected HeRs from among 470 HeR homologues in a BLAST search. We constructed plasmids for the expression of the selected HeR genes from genomic DNA by PCR or chemically synthesized (IDT). The HeR genes were obtained from *Thermococcus* sp. 2,319 × 1, *Halorhabdus tiamatea* SARL4B, *Mesotoga inferea*, *Degalogenimonas alkenigignes*, *Halolactibacillus*, *Salinibacterium xinjiangense*, *Demiquina* lutea, *Salinibacterium* sp. PAMC21357, *Streptomyces* sp. CC77, *Knoellia aerolata*, *Nocardioides dokdonensis*, *Actinobacteria* bacterium IMCC26103, and *Orinithinimicrobium pekingense*. The full-length HeRs with N-terminal His-tag were cloned into pKA001. The expression and purification of HeRs followed previous research^18^. The HeRs were expressed in *E. coli* strain UT5600. UT5600 cells transformed with HeRs were grown in LB broth overnight and 1% transferred to the new LB and cultured until the optical density at 600 nm reached 0.4 OD, and then induced with 1 mM IPTG (Applichem) and 5 ~ 10 μM all-*trans* retinal (Sigma) and incubated for 6 h at 35 °C. The *E. coli* cells expressing HeR were collected by low speed centrifugation. The cells were resuspended and disrupted by sonication (Branson sonifier 250) and the membrane fractions precipitated with ultracentrifugation at 35,000 rpm for 1 h. The membrane pellet of HeR was resuspended and solubilized with n-dodecyl-maltopyranoside (DDM). We used affinity chromatography and purified the His-tagged HeRs. To increase HeR purity, a diluted imidazole concentration was used with 150 mM NaCl, 50 mM Tris, and 0.02% DDM solution and repeated the centrifugation three times at 4,000 rpm for 30 min.

### Phylogenetic tree, sequence comparison, and prediction of secondary structure

The 48C12 HeR homologues were listed in the BLAST search, and the phylogenetic tree was constructed with Mega X^40^. Multiple sequence alignment was conducted using Clustal Omega^41,42^. The evolutionary history was inferred by the maximum likelihood method and JTT matrix-based model^40^. We randomly selected 13 HeRs from the BLAST results. Multiple sequence alignment was performed with these 13 randomly selected HeRs to find sequence singularity. We chose two HeRs for further analysis: the HeR from *Thermococcus* sp. 2,319 × 1 and the HeR from *Orinithinimicrobium pekingense.* One had a far-blue λmax among HeRs, and the other had a far-red λmax. Secondary structure prediction was performed using two HeRs. The secondary structure model was obtained with Protter^43^.

### Site-specific mutagenesis

Site-specific mutagenesis was performed using the mega-primer method^18^. We designed and constructed specific primers to create mutations on opsin genes of *Thermococcus* sp. 2,319 × 1 and *Orinithinimicrobium pekingense*. Mutation sites were selected through sequence comparisons among HeRs with λmax > 550 nm and HeRs with λmax < 540 nm (Fig. 2). We replaced the residues of GPPG (Gly-Pro-Pro-Gly) to FDEA (Phe-Asp-Glu-Ala), Q (Glu) to M (Met), and A (Ala) to S (Ser) for mutation on *Orinithinimicrobium* heliorhodopsin and replaced the residues of FDEA (Phe-Asp-Glu-Ala) to GPPG (Gly-Pro-Pro-Gly), M (Met) to Q (Gln), and S (Ser) to A (Ala) for mutation on *Thermococcus* sp. 2,319 × 1 heliorhodopsin. Double or triple mutants of candidate sites were also constructed to check large spectral shifts.

### Measurement of absorption spectra

Purified HeRs were prepared in 150 mM NaCl, 50 mM Tris, and 0.02% DDM solution at pH 7.0. UV/VIS spectroscopy was used to measure the absorption spectra of the purified HeRs with the Shimadzu UV–visible spectrophotometer (UV-2450).

### Protein topology and prediction of retinal-binding pocket region

Protein topology and the retinal binding pocket were predicted based on a previous structural study by Wataru Shihoya et al^24^. The topology prediction for *Ornithinimicrobium* heliorhodopsin was obtained with Protter^43^. We designed the chromophore and location of target amino acids of HeRs using SWISS-MODEL^44^. We built the 3D structure of NOH and NTH using the crystal structure of *Thermoplasmatales Archaeon* heliorhodopsin (PDB code: 6is6) as a template^24^. After obtaining the predicted structure, the chromophore and interaction of amino acids were visualized using PyMol.

## Supplementary information

Supplementary Figure S1
