# Supplementary material for: Color-tuning of natural variants of heliorhodopsin
Source: Sci Rep. 2021 Jan 13;11:854. doi: 10.1038/s41598-020-72125-0 (PMC7807009; doi:10.1038/s41598-020-72125-0)
Supplement: Supplementary file 1 — Supplementary Figure S1 [file 41598_2020_72125_MOESM1_ESM.docx]

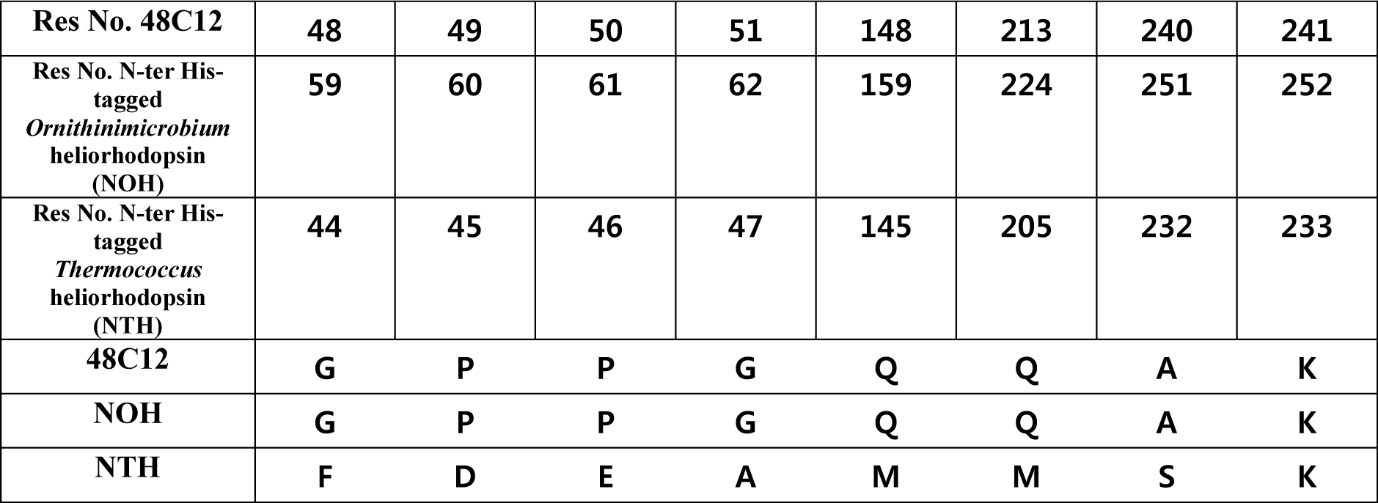


Figure S1. The panel indicated the amino acid sequence alignment of 48C12, N-ter His-tagged *Ornithinimicrobium* heliorhodopsin (NOH) and N-ter His-tagged *Thermococcus* heliorhodopsin (NTH).
